# Serotonin and the bone assessment

**Published:** 2014

**Authors:** M Carsote, V Radoi, A Geleriu, A Mihai, D Ferechide, D Opris, D Paun, C Poiana

**Affiliations:** * “Carol Davila” University of Medicine and Pharmacy, Bucharest, Romania; ** “C.I. Parhon” National Institute of Endocrinology, Bucharest, Romania

**Keywords:** serotonin, osteoporosis, CrossLaps, Osteocalcin, Alkaline Phosphatase

## Abstract

**Introduction:** Lately, the in vitro and in vivo studies on serotonin metabolism have been pointing its influence in bone health. Also, there are no particular recommendations in performing the serum serotonin assessment in order to evaluate the skeletal status.

**Aim:** We aimed to correlate the bone turnover markers and lumbar bone mineral density (BMD) with serotonin.

**Material and methods:** There is a cross-sectional study in Caucasian postmenopausal women. They were not diagnosed with carcinoid syndrome, or bone anomalies, and received no treatment (including antiresorptives). The following bone formation markers were performed: serum alkaline phosphatase (AP), serum osteocalcin (OC), and the bone resorption marker: serum CrossLaps (CL). Serum serotonin (high-pressure liquid chromatography), as well as central DXA (GE Prodigy) were assessed.

**Results:** 191 women of 57.1 years mean age were grouped according to DXA (WHO criteria). The linear regression analysis between serum serotonin and CL were not statistically significant (SS), between serotonin and OC was SS in the newly diagnosed osteoporosis group (N=40, r=0.4, p=0.03), between serotonin and AP SS was found in osteopenia group (N=88, r=0.24, p=0.03), with no changes when adjusting for age and BMI. The partial correlation between serotonin and BMD was not SS.

**Discussion:** The study raises the question of serotonin as a bone metabolism marker seeing that the results were not consistent. The main limit of our study was that we did not analyze the possible use of antidepressants to these women. Overall, this was a pilot study in clinical practice where few reports have been published, but still necessary, because the use of serum serotonin in current skeletal evaluation is still unclear.

## Introduction

The serotonin is a well-known brain neurotransmitter but during the last years, a great interest has been noticed in its actions over the bone. The dynamic of understanding the serotonin signaling has changed since 5-hydroxytryptamine with gut origin was found to regulate the bone loss via LRP-5 [**1**,**2**]. In vitro studies revealed that human osteoblasts and osteoclasts express tryptophan hydroxylase type 1, serotonin transporter and serotonin receptors (type 2A only in osteoblasts, type 1B in both osteoclasts and osteoblasts, and type 2B in precursors and mature osteoclasts), while selective serotonin reuptake inhibitors (SSRI), induce apoptosis of both types of cells [**3**]. Moreover, studies in female mice pointed bone microarchitecture changes of the distal femur as characterized by X-ray micro computed tomography analysis under the effect of antidepressants, probably by interfering with serotonin metabolism [**4**]. The clinical studies in literature found an increased risk of fracture based on high bone turnover markers and low bone mineral density in patients with depression and (SSRI) antidepressants [**5**]. The most important effect was due to the activation of 5-hydroxytriptamin receptors on bone (mainly on osteoclasts and osteoblasts) by using different pathways as endocrine or neural pathways [**6**]. The other observations on patients with depression pointed an increase of serum osteocalcin and decrease of serum resorption β-CTX marker after depression therapy with SSRI drugs [**7**]. The evidence between serotonin actions on bone raised the still unanswered question, as which is the exact place of serotonin assessment and if SSRI should be listed among the many causes of bone loss [**8**]. Some reports express a twofold fracture risk in SSRI users versus non-SSRI users but the risk is different with regard to the type of drugs, to the timing of therapy or the discontinuing of the medication [**9**].

The serotonin studies at different levels and the association with metabolism complications involve various observations. One study in 264 Japanese women found a correlation between fasting blood glucose and polymorphisms of the serotonin transporter-linked polymorphic region (5-HTTLPR) which is the main regulator of the transcriptional activity of serotonin [**10**]. In a report on 252 Greek subjects with type 2 diabetes, the S allele of 5-HTTLPR was associated with this glucose pathology [**11**]. The same type of connections was found on 234 type 2 diabetic patients with an increased risk of anxiety/depression in cases with 5-HTTLPR/rs25531 genotype [**12**]. The observations from Kansai Medical University refer to the plates that excessively release serotonin parallel to the renal function damage in diabetic subjects [**13**]. Another mechanism that involves serotonin in diabetes is, as proved in a rat model, the possible disturbances of insulin communication in the hypothalamus [**14**]. The anomalies were also found in adipocytes where their long-term exposure to high levels of serotonin induces insulin resistance [**15**]. The metabolic complications pathways are closely connected to the bone status via serotonin signaling. One relationship is established via leptin in serotoninergic brain signaling acting both on food regulation and on bone mass [**16**].

Our aim was to correlate the bone turnover markers or Dual Energy X-ray Absorptiometry (DXA) assessment with the levels of serum serotonin in postmenopausal women without a previous specific bone disease. 

## Material and Methods

This is a cross-sectional pilot original research study. We included Caucasian women in menopause who were not previously diagnosed with bone diseases as osteoporosis or Paget disease, etc. They were 40 years and older. We excluded the subjects previously treated for osteoporosis or for fragility fracture risk prevention as bisphosphonates, and also the patients previously or currently treated for bone metastases. Moreover, the women known with carcinoid disease or neuroendocrine tumors were not enrolled. 

Anamnesis was performed; the weight (in kilograms or kg) and the height (in meters or m) were measured in order to calculate the Body Mass Index (BMI) in kg/m2. The serum levels of calcium and phosphorus were assessed. The bone turnover markers were evaluated: the bone formation markers were serum alkaline phosphatase or AP (colorimetric assay) in Units/Liter (U/L), serum osteocalcin or OC (fotochemiluminescence assay) in nanogram/millimeter (ng/mL); the bone resorption marker was serum CrossLaps or CL in nanogram/millimeter (ng/mL). The serum serotonin was performed (high-pressure liquid chromatography). The normal values of the bone turnover markers were AP between 35 and 129 U/L, CL between 0.166 and 0.476 ng/mL, OC between 4.9 and 30.5 ng/mL. The normal serotonin values were between 80 and 450 ng/mL. All the subjects had a central DXA at least at two central sites (with a GE Prodigy device). This analysis was performed by using the data provided by lumbar spine DXA: bone mineral density (BMD) in g/cm2. The WHO criteria of osteoporosis were applied to the diagnosis of osteoporosis (T-score ≤ 2.5SD), osteopenia (T-score >-2.5SD and ≤-1), and normal DXA (T-score >-1) [17].

**Statistical analysis**

The studied parameters were expressed as mean, standard deviation, ranges. SPSS 21 (IBM C) was used to calculate bivariate and partial correlations (to adjust the effect of age and BMI) between serotonin and lumbar BMD. Linear regressions and bivariate correlations were calculated for serotonin - osteocalcin, serotonin - CrossLaps and serotonin - alkaline phosphatase and the results were the same up to the second decimal. Partial correlations for the adjustment of the effect of age and BMI were also calculated for these three relationships. A two sided alpha value of under 0.05 was considered statically significant (p<0.05). 

## Results

191 postmenopausal women were enrolled. The mean age at evaluation was 57.109 years. The mean BMI was 29.088 kg/m2. The values of the bone turnover markers were calculated (**Table 1**).

**Table 1:** The baseline characteristic of the entire cohort (number of subjects: N=191)

Parameters	Minimum	Maximum	Mean	Std. Deviation
age (years)	41	78	57.109	7.683
BMI (kg/m2)	18	64	29.088	6.205
serotonin (ng/mL)	23	393	159.98	69.019
CL(ng/ml)	0.03	1.6600	0.452	0.269
OC(ng/ml)	4.061	69.990	22.262	11.027

63 subjects had a normal DXA with a mean age of 53.269 years (**Table 2**).

**Table 2:** The baseline characteristic of the subjects with normal DXA (number of subjects: N=63)

Parameters	Minimum	Maximum	Mean	Std. Deviation
age (years)	41	66	53.269	5.355
BMI (kg/m2)	19	50	30.134	6.316
serotonin (ng/mL)	25	323	154.349	67.783
CL(ng/ml)	0.14	1.43	0.445	0.288
OC(ng/ml)	6.6	67.95	20.627	10.538
AP (U/L)	39	238	78.667	29.999

88 women had osteopenia (**Table 3**).

**Table 3:** The baseline characteristic of the subjects with osteopenia based on central DXA (number of subjects: N=88)

Parameters	Minimum	Maximum	Mean	Std. Deviation
age (years)	42	78	57.943	7.558
BMI (kg/m2)	18.5	64	29.228	6.334
serotonin (ng/mL)	23	393	166.465	74.625
CL(ng/ml)	0.03	1.66	0.464	0.281
OC(ng/ml)	6.84	69.99	23.004	11.352
AP (U/L)	28.4	153.67	78.841	23.586

40 patients were diagnosed with osteoporosis based on DXA and applying the WHO criteria (**Table 4**).

**Table 4:** The baseline characteristic of the subjects with osteoporosis (number of subjects: N=40)

Parameters	Minimum	Maximum	Mean	Std. Deviation
age (years)	44	78	61.325	8.422
BMI (kg/m2)	18	37	27.131	5.379
serotonin (ng/mL)	41	319	154.6	57.486
CL(ng/ml)	0.13	1.02	0.439	0.201
OC(ng/ml)	4.061	58.86	23.543	11.109
AP (U/L)	46	153.	79.512	21.974

The mean values of the serum serotonin were within the normal ranges: for the entire cohort (159.98 ng/mL), for subjects with normal DXA (154.349 ng/mL), for osteopenia group (166.465 ng/mL), and osteoporosis group (154.6 ng/mL). The higher value was registered in the women with osteopenia with no statistical significant difference between the three groups. The linear regression analysis between serum serotonin levels and the bone formation marker serum osteocalcin pointed a positive r-value for the entire studied population and for each of the three DXA groups (DXA normal, osteopenia, and osteoporosis). None of these results was statistically significant (**Table 5**).

**Table 5:** The linear regression between serotonin and osteocalcin (OC), CrossLaps (CL), and alkaline phosphatase (AP). The partial correlation between serotonin and lumbar BMD (DXA)

correlation	Serotonin - OC		Serotonin - CL		Serotonin - AP		Serotonin - BMD	
	r	p	r	p	r	p	r	p
all	0.07	0.4	0.05	0.53	0.07	0.35	0.02	0.77
all (adjusted for age and BMI)	0.06	0.43	0.05	0.52	0.08	0.31	0.03	0.97
normal DXA	0.08	0.56	-0.07	0.62	-0.17	0.19	-0.13	0.3
normal DXA (adjusted for age and BMI)	0.04	0.77	-0.01	0.96	-0.14	0.29	-0.14	0.3
osteopenia	0	0.99	0.05	0.66	0.24	0.03	0.14	0.,2
osteopenia (adjusted for age and BMI)	0	0.99	0.06	0.63	0.24	0.03	0.14	0.2
osteoporosis	0.24	0.19	0.4	0.03	0.18	0.29	0.15	0.34
osteoporosis (adjusted for age and BMI)	0.24	0.21	0.4	0.03	0.18	0.29	0.16	0.33

**Fig. 1:**
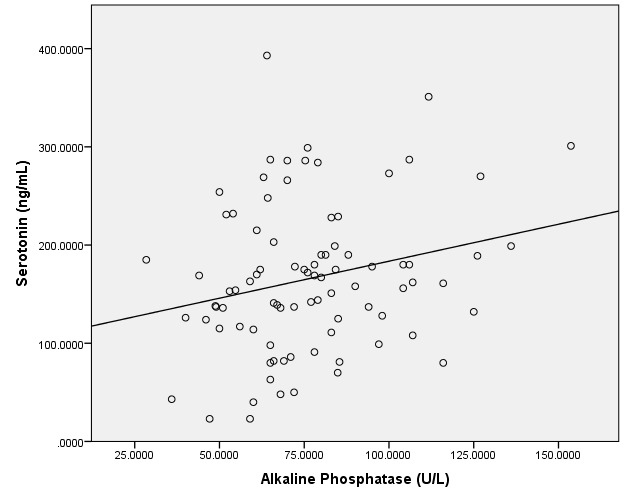
The linear regression between serotonin (ng/mL) and alkaline phosphatase (U/L) in subjects with osteopenia

The linear regression between serotonin and the resorption marker serum CrossLaps was positive, except for the normal DXA group. Statistically significant results were found in the subjects with osteoporosis (N=40), meaning r=0.4, p=0.03, with similar results when adjusting for age and BMI. The linear regression between serotonin, on one hand, and serum alkaline phosphatase, on the other hand, was positive, except for the women with normal DXA evaluation. The only statistically significant values were in the patients with osteopenia: r=0.24, p=0.03, with no changes when adjusting for age and BMI (**Fig. 1**).

The partial correlation between serotonin levels and lumbar BMD (DXA) was positive for all the patients, and for each of the groups with osteopenia, and osteoporosis, but no results had statistical relevance (**Table 5**).

## Discussions

This study represents an attempt to point out the place of the serum serotonin as a possible bone turnover marker. There are very limited similar data in literature, in this particular field of clinical practice involving the current bone evaluation in apparently normal subjects, meaning with no particular pathology related to the serotonin metabolism. 

Based on our observations, the partial correlation between serotonin and lumbar DXA was not statistically significant. From another point of view, the present analysis regarding the bone turnover markers showed statistically significant results between the levels of serotonin and CrossLaps in osteoporotic women, and between the levels of serotonin and alkaline phosphatase in osteopenic subjects. It seems that serum serotonin is a possible resorption marker, more useful in postmenopausal women with abnormal DXA results than in those with normal DXA. The clinical use of performing serotonin in order to obtain more information about the bone is still unclear. For example, studies in untreated patients with carcinoid syndrome could not find significant changes in bone turnover markers despite high levels of serum serotonin, and consecutively increased urinary 5-hydroxy indole acetic acid [**18**].

The present study has some limits. One of them is the limited number of patients with osteoporosis (N=40) but the total number of 191 subjects to whom both serotonin and bone evaluation were performed is relatively large compared to preexistent data in literature. Another is the fact that we did not focus on the subjects’ history regarding different types of medication, especially from the psychiatric area because we considered the peripheral levels of serotonin as the most useful marker to assess the complex serotonin metabolism in current clinical practice, regardless the interferences of its metabolism pathways. Moreover, our aim was to evaluate the use of serotonin assessment independently of depression and antidepressant drugs. Generally, it is known that in short term, SSRI administration increases the 5-hydroxy-tryptophan levels, but on long term, its levels decrease more than a half [**19**]. Another aspect is related to the fact that a depressed individual displays a lower BMD and higher bone resorption markers than non-depressed people, but the direct serotonin underlying mechanism is still unclear [**20**]. Some data in literature supported the idea of type 2 diabetes mellitus and obesity, linked to the serotonin metabolism, but in our study, the BMI influence was adjusted, with no significant changes of the results [**11**].

## Conclusion

This pilot study in a field with very few similarities in the current clinical non-psychiatric practice revealed some correlations between the levels of serum serotonin and the bone turnover markers, but none between the levels of serotonin and the bone mass density as provided by lumbar DXA in postmenopausal women. The exact place of serotonin in the skeletal health assessment is still a matter of debate.

**Conflict of interest**

The authors have nothing to disclose.
